# *Chrysanthemum indicum* and *Chrysanthemum morifolium*: Chemical Composition of Their Essential Oils and Their Potential Use as Natural Preservatives with Antimicrobial and Antioxidant Activities

**DOI:** 10.3390/foods9101460

**Published:** 2020-10-14

**Authors:** Fadia S. Youssef, Safaa Y. Eid, Elham Alshammari, Mohamed L. Ashour, Michael Wink, Mahmoud Z. El-Readi

**Affiliations:** 1 Department of Pharmacognosy, Faculty of Pharmacy, Ain Shams University, Cairo 11566, Egypt; fadiayoussef@pharma.asu.edu.eg (F.S.Y.); ashour@pharma.asu.edu.eg (M.L.A.); 2 Department of Biochemistry, Faculty of Medicine, Umm Al-Qura University, Makkah 21955, Saudi Arabia; syeid@uqu.edu.sa; 3 Department of Pharmacy Practice, College of Pharmacy, Princess Nourah bint Abdulrahman University, Riyadh 11671, Saudi Arabia; ejalshammari@pnu.edu.sa; 4 Department of Pharmaceutical Biology, Institute of Pharmacy and Molecular Biotechnology, Heidelberg University, 69120 Heidelberg, Germany; 5 Department of Biochemistry, Faculty of Pharmacy, Al-Azhar University, Assiut 71524, Egypt

**Keywords:** Asteraceae, antimicrobial, antioxidant, *Chrysanthemum*, essential oil, preservative

## Abstract

The composition of essential oils of *Chrysanthemum indicum* and *C. morifolium* were comparatively studied using both Gas Chromatography/Flame ionization Detector (GC/FID) and Gas Chromatography/Mass spectrometry (GC/MS) analyses. The antiviral activity was determined using a plaque reduction assay against three common viruses namely, herpes simplex type-1 (HSV-1), hepatitis A (HAV) and vesicular stomatitis virus (VSV). The antimicrobial activity was assessed using agar diffusion and microdilution methods and the minimum inhibitory concentration (MIC) values were determined. In addition, the anti-mycobacterial evaluation was carried out using the Alamar blue assay and the effect against *Helicobacter pylori* was investigated. The anti-trypanosomal activity was evaluated using the resazurin method. GC investigations revealed that camphor is the major constituent of both oils accounting for 36.69 and 14.56% in the essential oils from *C. indicum* and *C. morifolium*, respectively. *C. indicum* was biologically more active in all experiments; it exhibited a notable antitrypanosomal activity with an IC_50_ value equals 45.89 μg/mL and a notable antimicrobial activity versus *Streptococcus agalactiae* with a MIC value of 62.5 μg/mL. It also inhibited the replication of VSV with an IC_50_ value of 3.14 μg/mL. Both oils revealed antioxidant potential with IC_50_ values of 2.21 and 2.59 mg/mL for *C. indicum* and *C. morifolium,* respectively. This study provides evidence beyond the traditional use of both *Chrysanthemum indicum* and *C. morifolium* as anti-infective agents. Thus they could be used as spices in food and can be incorporated in different food products and pharmaceutical preparations as natural preservatives possessing antioxidant potential.

## 1. Introduction

Medicinal plants provide a large chemical library of secondary metabolites, which are widely used in phytotherapy for their antiviral, antimicrobial, anti-trypanosomal, anticancer and antioxidant activities [1]. Among them, essential oils are common in many aromatic plants. Researchers have continued to explore the chemical composition of the essential oils and their biological activities aiming to discover new bioactive and useful metabolites for the treatment of human and animal health conditions [2,3].

Based on traditional Chinese medicine and Ayurveda, the dried flower heads of many *Chrysanthemum* plants were widely used for the treatment of common cold [4], fever, migraine, conjunctivitis, eye irritation, hypertension, inflammation, ulcerative colitis, vertigo, ophthalmia with swelling as well as skin infections [5,6,7].

This genus, *Chrysanthemum* L. (*Dendranthema* (DC.)) belongs to the family Asteraceae, and comprises about 40 species, widely distributed in Asia, mainly in Mongolia, China and Japan, and eastern Europe. Nowadays, most of these plants are cultivated as ornamentals in the whole world [8]. *C. morifolium* Ramat. (Jiu Hua) and *C. indicum* L. (Ye Jiu Hua) are common in China [9]. They are generally perennial herbs or subshrubs with aromatic alternate, lobed or serrate leaves. Capitula are corymbs or solitary with yellow, 5-lobed corolla disc florets meanwhile ray florets are white, pink or yellowish and achenes are obvoid with 5–8 ribs [10,11].

Thorough phytochemical and biological investigations of both plants led to the isolation and identification of many bioactive secondary metabolites represented mainly by flavonoids, steroids and terpenoids [12,13,14,15,16,17]. These plants exhibit strong antibacterial, antiviral, antioxidant, antihypertension, anti-inflammatory and immunomodulatory activities [18,19,20,21,22,23,24,25,26].

The chemical compositions of *C. indicum* and *C. morifolium* essential oils have been previously studied. Camphor, borneol, camphene, α-pinene, *p*-cymene and 1,8 cineole are the major constituents of the oils [27,28]. In spite of the significant biological importance of these plants, their essential oils were not fully investigated, only the antimicrobial activity had been studied [28].

In this study, a detailed comparative study regarding the chemical composition and anti-infective properties of *C. morifolium* and *C. indicum* essential oils was presented. The biological activities of the essential oils against a wide range of microbes and viruses were investigated. Moreover, microorganisms of food and clinical interest have been selected as target for the antimicrobial evaluation to further consolidate the rationale beyond the traditional use of both plants for the first time as anti-infective agents aiming to use these plants and their oils as natural spices and preservatives both in food and pharmaceutical preparations.

## 2. Materials and Methods

### 2.1. Plant Material

The flower heads of *Chrysanthemum morifolium* Ramat. and *C. indicum* L. (family Asteraceae) were commercially obtained from China. DNA barcoding technique was used to authenticate the plants and it was confirmed morphologically at the Botanical Garden, Heidelberg University. The flower materials are stored at the Department of Biology, IPMB, Heidelberg University under the numbers of P6851 and P6852, respectively.

### 2.2. Chemicals, Reagents and Strains

All cell culture materials and media were obtained from Gibco^®^ (Invitrogen, Karlsruhe, Germany) and Greiner Labortechnik^®^ (Frickenhausen, Germany). Chemicals and reference drugs were bought from Sigma^®^ (Sternheim, Germany). The solvents with analytical grade were procured from Merck^®^ (Darmstadt, Germany). All the tested microorganisms were provided by Medical Microbiology Lab., Hygiene Institute, Heidelberg University, Germany. *Mycobacterium tuberculosis* (RCMB 010126) strain was obtained from RCMB, Al-Azhar University, Cairo, Egypt and *Helicobacter pylori* ATCC 43504 strain from the American Type Culture Collection.

### 2.3. Essential Oils Preparation

The air-dried flower heads of *C. morifolium* and *C. indicum* were hydro- distillated by Clevenger-type apparatus (Faculty of Bioscience, Heidelberg University glassware Lab, Heidelberg, Germany) for 6 h. Dehydrated essential oils (EO) were obtained by using anhydrous Na_2_SO_4_ to yield 0.16% and 0.18% dry weight, respectively. The essential oils were stored in amber glass with stopper vials at −30 °C until analysis.

### 2.4. GC/FID and GC/MS Analyses

GC 5890 II (Hewlett-Packard GmbH, Homburg, Germany) coupled with quadrupole mass spectrometer (SSQ 7000, Thermo-Finnigan, Bremen, Germany) was used for the identification of the essential oil compounds. Instrumental settings were *Gas*: ^4^He (2 mL/min); *Temp*: T_0_/45 °C, T_2_/isothermal, T_1_/300 °C, T_2_/isothermal, detector/300 °C, and injector/250 °C, *Split ratio*: 1:20. Varian 3400 (Varian GmbH, Darmstadt, Germany) was used for GC analyses with OV-5 fused bonded column (Ohio Valley, OH, USA) and a Flame Ionization Detector (FID) for quantitative determination of the components. Mean of 3 independent runs was used to calculate areas under the peaks (AUP) using an SRI Instrument (Torrance, CA, USA) and the total area was considered as 100%. Wiley Registry of Mass Spectral Data 8th edition, National Institute of Standards and Technology (NIST) Mass Spectral Library (December 2005), and the literature were used to compare and identify the active constituent of spectral data [29,30].

### 2.5. Cytotoxic Assay

Cytotoxicity was determined in triplicates using MTT cell viability assay [31]. A total of 2 × 10^4^ cells/well of both cell lines CCL-81 (Vero) (ATCC 43255) and human HL-60 (human myeloid cells) (ATCC CCL-240) were incubated for 24 h at 37 °C with a humidified atmosphere of 5% CO_2_ in complete DMEM media and in complete RPMI media, respectively. The EOs were dissolved in DMSO, the final concentration of DMSO was <0.01% and the absorbance values of DMSO were subtracted from the sample absorbance. Several concentrations of EO (1 to 1000 μg/mL) and doxorubicin (positive control) were applied individually to the cells and incubated for further 24 h with the same conditions. The light absorption of formazan crystal that formed after incubation for 4 h with MTT (0.5 mg/mL) and solubilized by DMSO (200 µL) was measured at 570 nm using a Tecan^®^ Safire II Reader (Männedorf, Switzerland). The percentage of cell viability of three independent experiments was calculated as follows: cell viability (%) = (OD of treated cells − OD of DMSO treated)/(OD of control cells − OD of DMSO treated) × 100%.

### 2.6. Antiviral Assay

Plaque reduction assay was used to determine the antiviral activity of EO as previously described [32]. Briefly, Vero cells (CCL-81) were cultivated for 24 h at 5% CO_2_ and 37 °C. The cells were inoculated for 1 h with individual viruses; herpes simplex type-1 virus HSV-1 (ATCC VR-1493), hepatitis A virus HAV (ATCC VR-1357) or vesicular stomatitis virus VSV (ATCC VR-1238) (1 × 10^1^‒10^7^ plaque-forming unit PFU/mL). The infected cells (2 × 10^3^ PFU) were washed and incubated with several concentrations of EO as well as acyclovir (positive control) for 1 h. Then, cells were overlaid with nutrient agarose and incubated for 72 h. Formaldehyde (10%) in phosphate-buffer solution (pH 7.3) was used to fix cells for 1 h. Crystal violet, 0.5% in 20% ethanol, was used to stain cells and then plaques were counted. The percentage of viral inhibition was calculated as [1 − (V*d*/V*c*)] × 100, where V*d* and V*c* are the number of plaques in the presence and absence of test samples.

### 2.7. Antimicrobial Assay

Gram positive bacteria: Bacillus subtilis (ATCC 6051), Staphylococcus aureus (ATCC 29213), Staphylococcus capitis (ATCC 35661), Staphylococcus epidermidis (ATCC 14990), Streptococcus agalactiae (ATCC 27956), Streptococcus pyogenes (ATCC 12344), Gram negative bacteria: Escherichia coli (ATCC 25922), Pseudomonas fluorescens (ATCC 13525), Salmonella typhimurium (ATCC 14028), Shigella flexneri (ATCC 700930), and fungi: Aspergillus fumigatus (ATCC 1022), Candida albicans (ATCC 90028), Geotrichum candidum (ATCC 12784), Syncephalastrum racemosum (ATCC 14831), and MDR (multidrug resistance) isolates were used to evaluate the antimicrobial activity of EOs. Bacteria and fungi were incubated for 24 h at 37 °C in Columbia media plus 5% sheep blood and for 48 h at 25 °C in CHRO-Magar^®^ Candida, respectively (BD, Heidelberg, Germany). The agar diffusion method was used to determine the sensitivity of tested microbes to the tested EO. Pathogens 1 × 10^6^ CFU/mL saline were incubated with Mueller Hinton agar as previously reported [33]. Pasteur pipette was used to cup wells (6 mm diameter). The EO (3.2 mg/mL), DMSO, and reference antibiotics (ampicillin, gentamycin, and clotrimazole) were applied to the experimental set. The clear zones surrounding the wells indicated the growth inhibition and the average diameters of triple wells were recorded. Micro-broth dilution method was used for the assessment of the MIC (minimum inhibition concentration) and MMC (minimum microbicidal concentration) of tested EO [34]. Several concentrations of EO were incubated with bacteria (5 × 10^5^ CFU/mL Mueller Hinton broth) for 24 h at 37 °C and fungi (5 × 10^5^ CFU/mL Sabouraud Dextrose broth) for 48 h at 25 °C in a 96-well plate and reference antibiotics. The MMC of EO was determined as the lowest concentration of EO that completely killed the microbe.

### 2.8. Anti-Mycobacterial Assay

Alamar blue assay (MABA) was used to determine the anti-TB activity of EO. *Mycobacterium tuberculosis* (RCMB 010126) strain was cultivated in complete 7H9-S medium in the dilution of 1:20. The inoculum (100 μL) was incubated for 7 days at 37 °C with and without several concentrations of EO and isoniazid (positive control) (100 μL) in 96-well plate. Following this, cells were incubated for further 24 h with alamar blue solution (30 μL). The alamar blue interacted with bacteria and changed from oxidized form (blue) to reduced form (pink). The color measured spectrophotometry and the inhibition % was calculated from the equation = 1 − (mean of test well/mean of blank wells) × 100. The lowest concentration of EO that prevents the color change is considered as MIC [35].

### 2.9. Anti-Helicobacter Pylori Assay

*Helicobacter pylori* (ATCC 43504) (100 μL) of 20% (*v/v*) bacterial suspensions were incubated with Mueller–Hinton broth for 24 h at 37 °C with 100 μL of several concentrations of EO as well as clarithromycin as positive control in 96 well plate. Then, the MTT protocol was applied using the abovementioned cytotoxicity assay [35].

### 2.10. Anti-Trypanosomal Assay

Trypanocidal activity of EO was determined using resazurin dye as previously described [36]. *Trypanosoma b. brucei* TC221 (1 × 10^4^ cells/well) were incubated in complete Baltz medium with several concentrations of EO and diminazene (positive control) for 24 h at 5% CO_2_ and 37 °C. Following this, cells were incubated with resazurin (10 µL) for further 24 h. The absorbance at λ of 492 and 595 nm was measured by Tecan^®^ plate reader and the IC_50_ was calculated [37].

### 2.11. Antioxidant Determination

#### 2.11.1. Diphenyl-1-Picrylhydrazyl Scavenging Capacity Assay

The antioxidant potential was determined adopting the method which was reported previously by Nenadis et.al and Youssef et.al [38,39]. Briefly, 200 µL of samples at various concentration ranging between (1–100,000 μg/mL) and 3.8 mL of 60 µg/mL of DPPH methanol solution were mixed with each other. The mixture representing the reaction was maintained in the dark for 30 min at room temperature then the absorbance at 517 nm was measured. Quercetin was used as a positive control. DPPH•: free radical scavenging activity was assessed using the equation presented below:

% Inhibition = [Ac − As/Ac] × 100,

where Ac: absorbance of control; As: absorbance of sample.

#### 2.11.2. Superoxide Radical Scavenging (SORS) Activity

The ability of the *C. indicum* and *C. morifolium* oil to scavenge superoxide anion radicals was evaluated according to the previously reported method [40]. In brief, several concentrations of oils and quercetin (used as a positive control) were added to SO generated solution (1 mL of Tris–HCL buffer (16 mM, pH-8), 1 mL of nitroblue tetrazolium NBT (50 µM), 1 mL of Nicotinamide adenine dinucleotide (NADH) (78 µM) solution and 1 mL of PMS (phenazine methosulfate) (10 µM). Incubation was done for 5 min at 25 °C and then the absorbance was measured at 560 nm in spectrophotometer. The percentage inhibition of O_2_^•-^ generation was calculated as follows:

% Inhibition = [Ac − As/Ac] × 100,

where Ac: absorbance of control; As: absorbance of sample.

#### 2.11.3. Hydroxyl Radical Scavenging (HRS) Assay

Deoxyribose degradation (HRS) assay was used to determine the ability of *C. indicum* and *C. morifolium* oils to scavenge hydroxyl radical according to the previously reported method [41]. Equal volumes of several concentrations of essential oils or quercetin (used as a positive control) were mixed with working solution of 28 mM 2-deoxy-2-ribose (2-DR), 1.04 mM EDTA, 200 µM FeCl_3_, and 1.0 mM H_2_O_2_. After the incubation period (1 h at 37 °C) thiobarbituric acid (TBA) and 2.8% trichloroacetic acid (TCA) (1:1 *v/v*) were added to the reaction mixture and incubated at 100 °C for 20 min. Absorbance was measured at 540 nm against a blank using S Jenway^®^ 6800 UV/VIS spectrophotometer (Essex, UK). The percentage inhibition of HO^•−^ generation was calculated as follows:

% Inhibition = [Ac − As/Ac] × 100,

where Ac: absorbance of control; As: absorbance of sample.

### 2.12. Statistical Analysis

All the assays were performed in triplicate and in three independent times. Data were expressed as the mean ± SD. The dose response curves, graphs were drawn and IC_50_ were calculated by four parameter logistic equation, and student’s *t*-test and/or the Kruskal–Wallis test were carried out by GraphPad Prism^®^ 8.02 (GraphPad Software, Inc., San Diego, CA, USA). The *p* value < 0.05 was considered a significance difference between comparison groups.

## 3. Results and Discussion

### 3.1. Volatile Oil Constituents of Chrysanthemum indicum and C. morifolium Flower Heads

**Figure 1:**
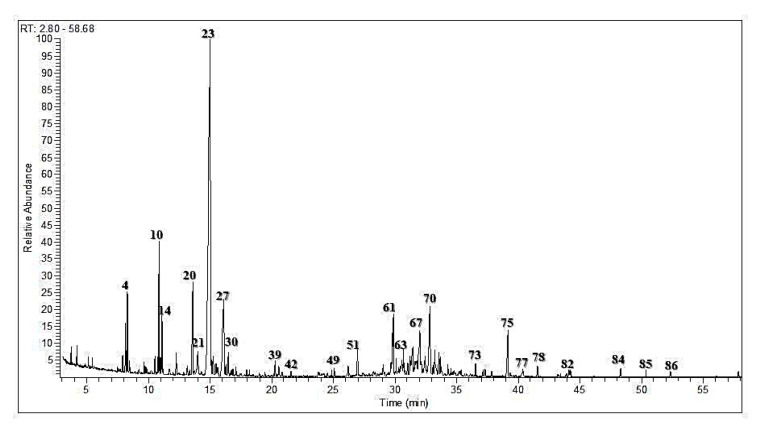
Gas Liquid Chromatography (GLC) profile of the volatile constituents in the essential oil of *C. indicum* flower heads.

**Figure 2:**
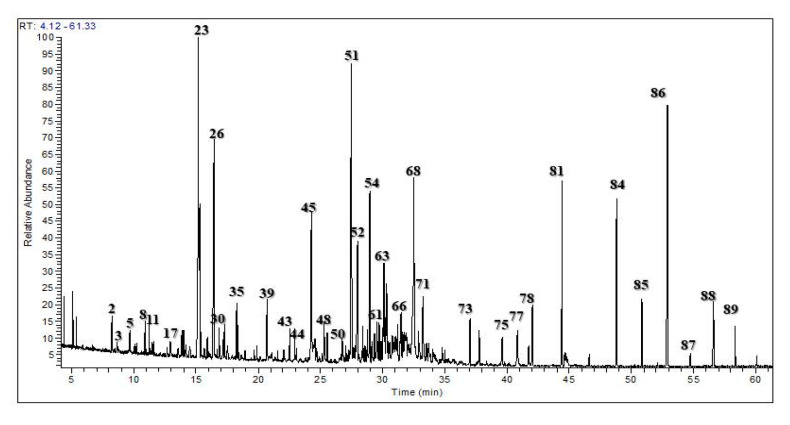
Gas Liquid Chromatography (GLC) profile of the volatile constituents in the essential oil of *C. morifolium* flower heads.

**Figure 3:**
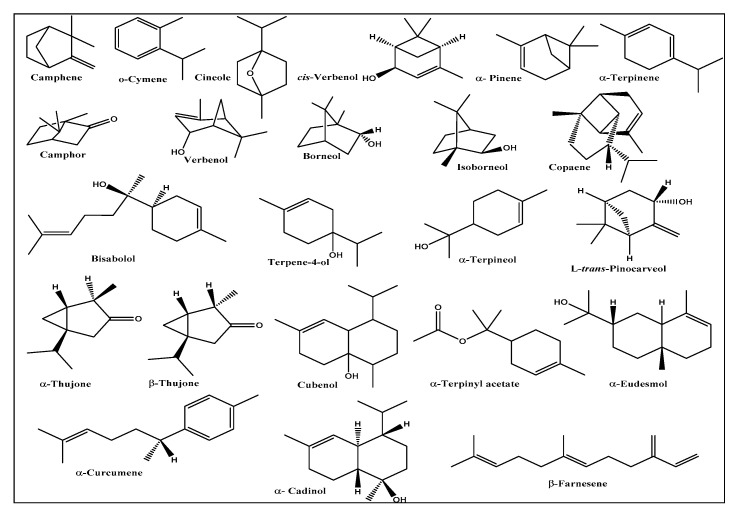
Major constituents of *C. indicum* and *C. morifolium* essential oils.

**Figure 4:**
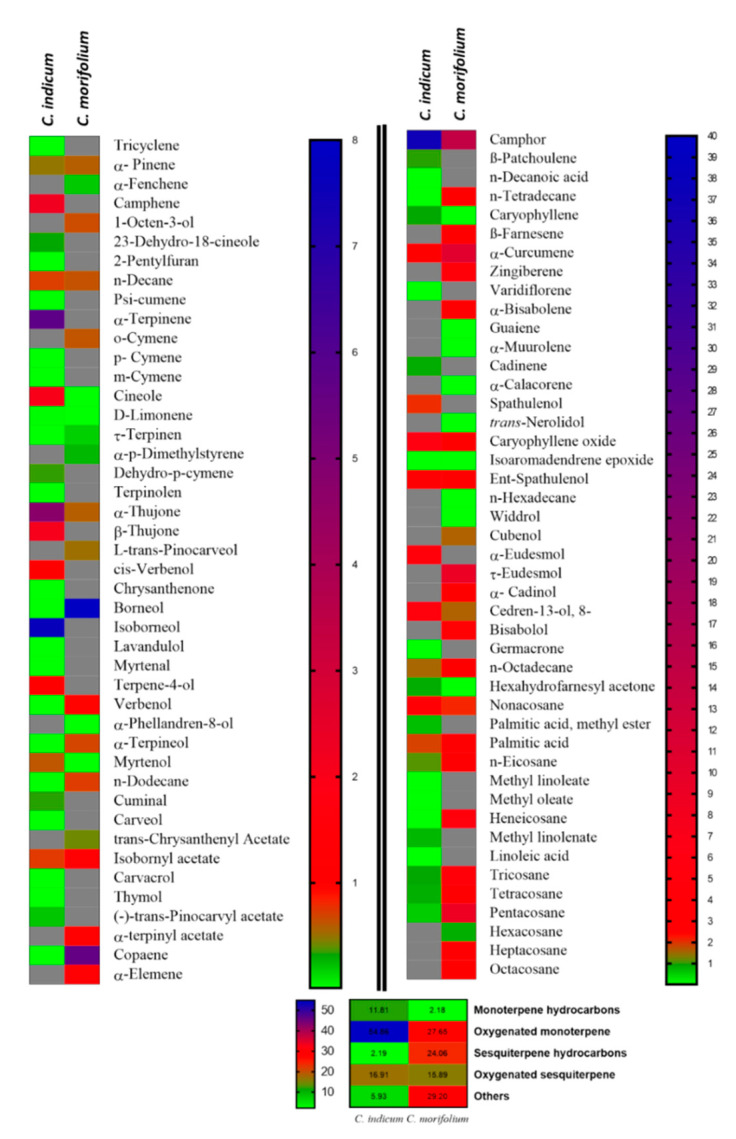
A heatmap comparison based on abundance of individual component of *C. indicum* and *C. morifolium*; gray color indicated non-detectable levels of identified component.

The essential oils from *Chrysanthemum indicum* and *C. morifolium* flower heads were comparatively studied using both GC/FID and GC/MS analyses. The aromatic essential oil from *C. indicum* flower heads has a blue color (0.16% *v/w* yield), whereas the aromatic oil from *C. morifolium* flower heads is greenish blue (yield 0.18% *v/w*). GC profiling revealed the presence of 89 metabolites representing 91.70 and 98.98% of the total essential oil composition of *C. indicum* and *C. morifolium* flower heads, respectively (Figure 1 and Figure 2). Sixty-four compounds were tentatively identified in *C. indicum* with camphor (36.69%) as the major constituent followed by isoborneol (7.64%), α-terpinene (5.73%), and caryophyllene oxide (5.46%). For *C. morifolium* fifty-five compounds were determined; similar to *C. indicum* camphor (14.56%) represented the prevailing compound followed by curcumene (10.50%), *τ*-eudesmol (8.92%), pentacosane (8.65%), borneol (7.95%) and copaene (5.61%) (Table 1). The major constituents present in both oils are illustrated in Figure 3. Apparently, oxygenated monoterpenes, represented mainly by camphor predominate in both oils. Moreover, monoterpenes and oxygenated sesquiterpenes exist in considerable amounts in the essential oil of *C. morifolium*, but in minor abundance in *C. indicum* oil. In contrast, monoterpenes form only 2.18% of the total identified oil components of *C. morifolium* essential oil. The differences in the composition of both oils were further illustrated using the heat map analysis, which is a type of graphical cluster analysis to better visualize the differences (Figure 4). The conditional formatting was applied using color code for each value. Dark grey coded for “non-detected compound”, green “for low levels content” from 0.01 to 0.9% meanwhile red coded from 1 to 8%, and blue for compounds showing % above 8%. Noteworthy to mention that the chemical composition of the essential oil obtained from the flowers of *C. morifolium* growing in Nigeria is quite different; its main constituent of the volatile oil is *cis* chrysanthenyl acetate (21.6%) followed by octadecanoic acid (19.5%) and borneol (15.5%) [42]. However, the essential oil obtained from the Korean *C. indicum* flower heads revealed nearly the same major constituents in which camphor represents the major component [43]. This greatly highlighted the impact of geographical distribution on the volatile oil compositions.

### 3.2. Antiviral Activity of Essential Oils of Chrysanthemum Indicum and C. morifolium Flower Heads

**Figure 5:**
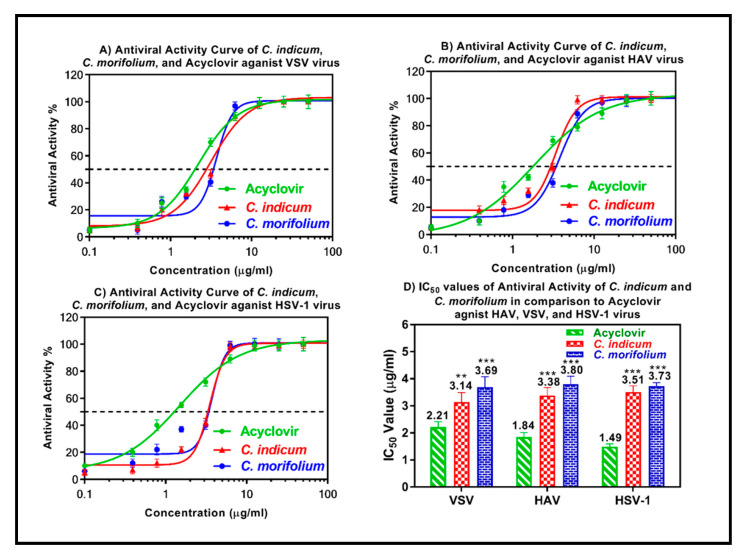
Antiviral activity of the essential oils obtained from *C. indicum* and *C. morifolium* flower heads versus vesicular stomatitis virus (VSV) (**A**), hepatitis A (HAV) (**B**) and herpes simplex type-1 (HSV-1) (**C**) using acyclovir as a standard antiviral drug. The means with different stars are significantly different (*p* < 0.05).

The antiviral activity of the essential oils obtained from the flower heads of *C. indicum* and *C. morifolium* was assessed using the plaque reduction assay on Vero cells (CCL-81). Nothing was previously reported in literature about the antiviral potential of the oils of interest. However, it was interesting to us that our previous work on aqueous extracts of both plants showed no activity against both hepatitis B virus (HBV), and bovine viral diarrhea virus (BVDV) [44]. Generally, most of the oils derived from the plant origin are evaluated against HSV-1 or rhinovirus. No reports could be traced regarding evaluation most of the oils against HAV or VSV. Both oils lack measurable cytotoxicity on Vero cells at the selected doses with IC_50_ values > 100 μg/mL being 125.81 and 145.35 μg/mL for *C. indicum* and *C. morifolium* oils, respectively as shown by the MTT assay. However, both oils in general and the *C. indicum* essential oil in particular, showed substantial antiviral potential in a dose dependent manner against VSV, HAV and HSV-1 as demonstrated in Figure 5. However, VSV was more sensitive to the antiviral activity of the *C. indicum* essential oil comparable to HAV and HSV-1 showing IC_50_ values of 3.14, 3.38 and 3.51 μg/mL towards the three previously mentioned viruses, respectively. It is worthy to mention that the activity of the *Chrysanthemum* oil is more potent than both clove and Eucalyptus oils which have a potent antiviral activity against HSV-1 [45]. Standard antiviral drug, acyclovir, displayed IC_50_ values of 2.21, 1.84 and 1.49 μg/mL towards VSV, HAV and HSV-1, respectively. The substantial antiviral activity of *C. indicum* essential oil could be interpreted in virtue of the presence of camphor, isoborneol and other major constituents. Camphor was previously reported to exert a remarkable virucidal potential versus herpes simplex virus-1 [46]. Additionally, isoborneol was previously reported to inhibit herpes simplex virus-1 replication via a specific inhibition in the glycosylation of its polypeptides exerting no cytotoxic effect on Vero cells (CCL-81) [47]. It is noteworthy to highlight that evaluation of cytotoxicity is crucially important in the assessment of the antiviral activity as beneficial oil should selectively inhibits viral replication, growth and other process with no or rare effects on different cellular metabolic process [48]. In general, lipophilic secondary metabolites interfere with the lipid membrane envelope of viruses. Therefore, they are not very specific and usually attack free viral particles and not intracellular viruses [49].

### 3.3. Antimicrobial Activity of C. indicum and C. morifolium Essential Oils

The antibacterial activity of essential oils obtained from the flower heads of *C. indicum* and *C. morifolium* was evaluated in vitro using the agar diffusion method against different standard Gram-positive and Gram-negative bacteria via measuring the mean diameter of inhibition zones (DIZ) and determining the minimum inhibition concentrations (MIC) values as well. Both oils exerted a moderate antibacterial activity against the tested Gram-positive bacteria with *C. indicum* essential oil being more potent comparable to *C. morifolium* oil displaying MIC of 62.5 µg/mL against each of *Bacillus subtilis*, *Streptococcus agalactiae* and *Streptococcus pyogenes.* However, both oils exerted weak activity versus the examined Gram-negative bacteria and fungal strains with MICs > 500 µg/mL. The mean diameter of inhibition zones (DIZ) and the minimum inhibition concentrations (MIC) of both oils versus all the tested bacterial and fungal strains are illustrated in Table 2. The results reported herein were in accordance with previously reported data for the essential oils from many *Chrysanthemum* species that exhibited antibacterial and antifungal potential versus many fungal and bacterial strains [28,50]. *C. indicum* essential oil flower heads growing in Korea exhibited significant antimicrobial potential versus all tested oral bacteria (showing MIC values between 0.1 and 1.6 mg/mL and Minimum Bactericidal Concentrations (MBCs) between 0.2 and 3.2 mg/mL) [51]. The existence of considerable amounts of monoterpenes and oxygenated monoterpenes could greatly explain the high potency versus Gram-positive bacteria via diffusing and destabilizing the structure of cell membrane. Thus, terpenoid rich essential oils are more effective against Gram-positive bacteria than to Gram-negative ones. Besides, a synergism could exist between major and minor compounds might probably contribute to the moderate antibacterial effect [52,53,54].

### 3.4. Anti-Mycobacterial and Anti-Helicobacter Pylori Activity of the Essential Oils

**Figure 6:**
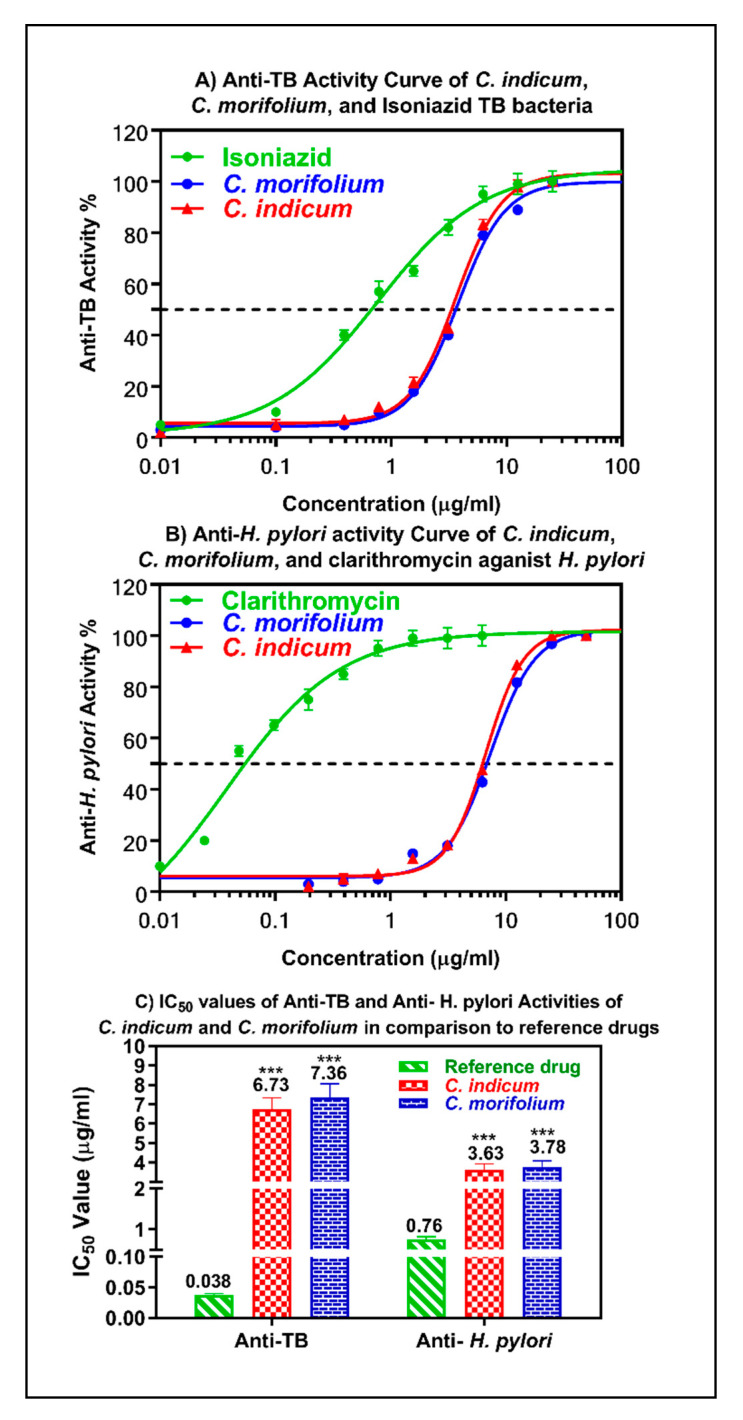
Anti-mycobacterial and anti-*Helicobacter pylori* activity of the essential oils obtained from *C. indicum* and *C. morifolium* flower heads versus reference drugs. The means with different stars are significantly different (*p* < 0.05).

Additionally, both oils showed a substantial anti-mycobacterial activity against *M. tuberculosis* with IC_50_ values of 6.73 and 7.36 µg/mL for *C. indicum* and *C. morifolium,* respectively, albeit lower than the positive control isoniazid (IC_50_ = 0.038 µg/mL). Nothing was previously reported in literature about the anti-mycobacterial and anti-*Helicobacter pylori* of the oils. Furthermore, both oils exhibited a good activity against *Helicobacter pylori* with IC_50_ values of 3.63 and 3.78 µg/mL for *C. indicum* and *C. morifolium,* respectively approaching that of clarithromycin (Figure 6). These results represent very good candidates for further studies taking into consideration that very limited work was done on the anti-*Helicobacter pylori* (only MICs were calculated and no IC_50_ could be found) [55,56]. The observed anti-mycobacterial and anti-*Helicobacter pylori* potential of the essential oils is mainly attributed to the presence of terpenes and fatty acids that account for the lipophilicity and greater penetration through the cell membrane triggering a massive disturbance in the oxidative phosphorylation and the electron transport chain as well resulting in a serious interference in the production of energy with concomitant evolution of auto-oxidation and peroxidation degradation compounds and bacterial lysis [35,57].

### 3.5. Anti-Trypanosomal Activity of Essential Oils

**Figure 7:**
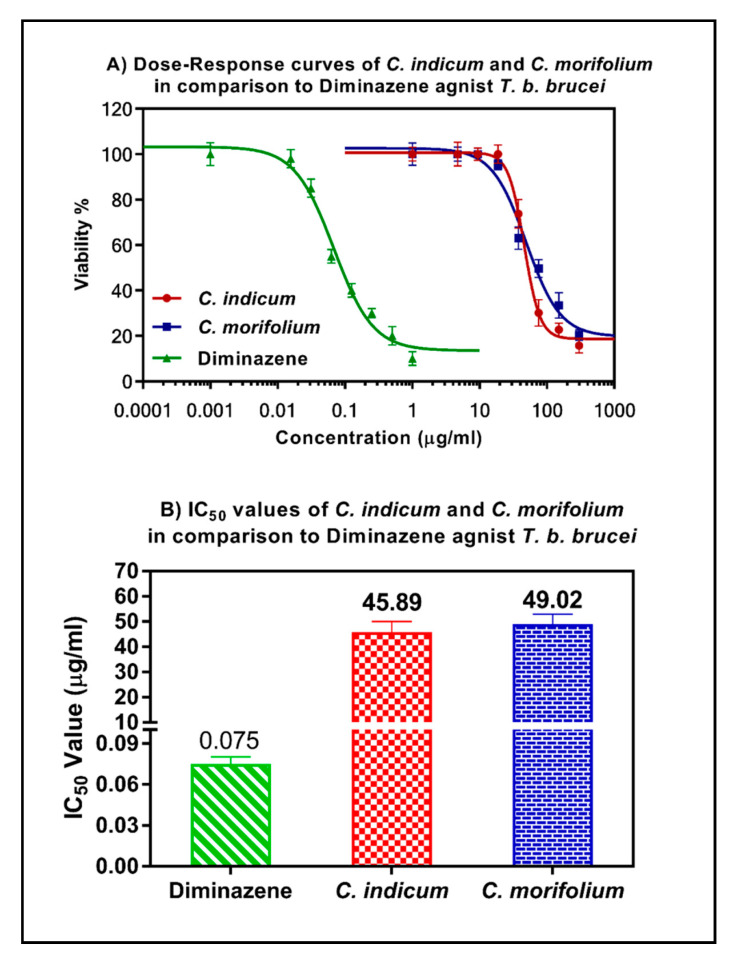
Anti-trypanosomal activity of the essential oils obtained from *C. indicum* and *C. morifolium* flower heads versus reference drugs.

Both essential oils showed a moderate anti-trypanosomal activity with IC_50_ values of 49.02 and 45.89 μg/mL, albeit lower than diminazene (positive control; IC_50_ = 0.075 μg/mL) (Figure 7). The selectivity indexes of the two oils were calculated as 1.8 and 1.5, respectively, indicating low selectivity against trypanosomes compared to their cytotoxicity versus HL-60 cells (88.24 and 68.84 μg/mL, respectively). It is worthy to highlight that nothing was traced in literature regarding the assessment of the anti-trypanosomal potency of both essential oils. However, it was noticed that both methanol and chloroform extracts of the same plants showed higher potency against *T. brucei*. *C. indicum* showed IC_50_s of 16 and 15.3 μg/mL for chloroform and methanol extracts, respectively [44]. In addition, this activity is better than our previously tested *Citrus* oils which exhibited lower activity with IC_50_ value = 60–72 μg/mL [33,58]. The activity of both oils is caused by their lipophilic terpenes which effectively interact with membrane lipids and proteins causing membrane disruption and ultimate cell death [59].

### 3.6. Antioxidant Activity of Essential Oils

**Figure 8:**
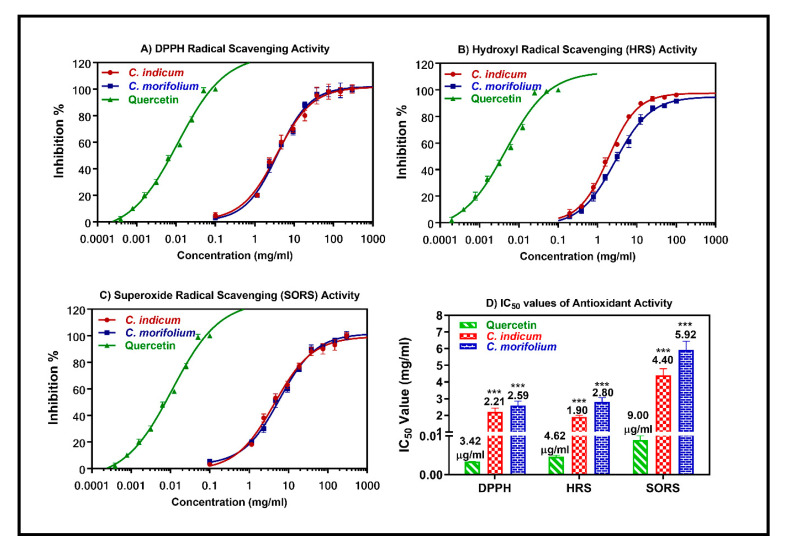
Antioxidant activities of the essential oils obtained from *C. indicum* and *C. morifolium* flower heads versus quercetin as a reference drug in DPPH (**A**), hydroxyl radical scavenging (HRS) (**B**), and superoxide radical scavenging (SORS) (**C**) assays. The means with different stars are significantly different (*p* < 0.05).

The antioxidant activity was assessed using DPPH, HRS, and SORS assays. The essential oils obtained from the flower heads of *C. indicum* and *C. morifolium* revealed substantial antioxidant potential with IC_50_ values of 2.21 and 2.59 mg/mL, in the DPPH assay, respectively, 1.90 and 2.89 mg/mL in HRS assay whereas they showed IC_50_ values of 4.40 and 5.92 mg/mL in SORS assay, respectively (Figure 8). The antioxidant activity can be interpreted by the synergistic interactions between all of the essential oil components in addition to their action as pro-oxidants. Inhibition of free radical chain reaction, destruction of peroxides, prompt clearance of free radical and hydrogen are familiar among the mode of action of many essential oils and thus could be the probable mode of action by which the tested oils exerted their effect.

## 4. Conclusions

Both essential oils exhibited antimicrobial, antiviral, anti-mycobacterial, anti-*Helicobacter pylori*, anti-trypanosomal and antioxidant activities with *C. indicum* being more effective. This study provides for the first time scientific consolidation beyond the traditional use of both *Chrysanthemum indicum* and *C. morifolium* as anti-infective agents. Thus, they could be used as spices in food and can be incorporated in different food products and pharmaceutical preparations as natural preservatives possessing antioxidant potential. However, further studies should be conducted to test their efficacy against other life-threatening microorganisms comprising the novel coronavirus (COVID-19) that threatens the worldwide. Moreover, in vivo studies are greatly recommended to further confirm the obtained results that should be proceeded by toxicity tests to guarantee the safety of the essential oils.

## Figures and Tables

**Table 1:** Volatile oil compositions of *C. indicum* and *C. morifolium* flower heads.

Compound		RI		Content [%]		Identification Method
		Cal.	Rep.	*C. indicum*	*C. morifolium*	
1.	Tricyclene	904	905	tr	-	MS, RI
2.	α- Pinene	917	917	0.49	0.57	MS, RI, AU
3.	α-Fenchene	930	934	-	0.20	MS, RI
4.	Camphene	933	933	2.21	-	MS, RI, AU
5.	1-Octen-3-ol	961	964	-	0.63	MS, RI
6.	2,3-Dehydro-1,8-cineole	972	972	0.32	-	MS, RI
7.	2-Pentylfuran	975	972	tr	-	MS, RI
8.	*n*-Decane	1000	1000	0.67	0.61	MS, RI
9.	Psi-cumene	1002	1004	tr	-	MS, RI
10.	α-Terpinene	1006	1006	5.73	-	MS, RI, AU
11.	*o*-Cymene	1008	1006	-	0.60	MS, RI
12.	*p*-Cymene	1009	1006	tr	-	MS, RI
13.	*m*-Cymene	1011	1010	tr	-	MS, RI
14.	Cineole	1015	1015	2.02	tr	MS, RI, AU
15.	D-Limonene	1017	1017	0.02	tr	MS, RI, AU
16.	*τ*-Terpinene	1046	1046	tr	0.18	MS, RI, AU
17.	α-*p*-Dimethylstyrene	1070	1070	-	0.26	MS, RI
18.	Dehydro-*p*-cymene	1070	1071	0.35	-	MS, RI
19.	Terpinolene	1075	1075	tr	-	MS, RI
20.	*α*-Thujone	1082	1087	4.73	0.57	MS, RI, AU
21.	*β*-Thujone	1093	1097	2.07	-	MS, RI, AU
22.	L-*trans*-Pinocarveol	1119	1119	-	0.51	MS, RI
23.	Camphor	1120	1124	36.69	14.56	MS, RI, AU
24.	*cis*-Verbenol	1126	1131	0.97	-	MS, RI, AU
25.	Chrysanthenone	1133	1131	tr	-	MS, RI
26.	Borneol	1136	1130	tr	7.95	MS, RI, AU
27.	Isoborneol	1149	1154	7.64	-	MS, RI, AU
28.	Lavandulol	1152	1152	tr	-	MS, RI
29.	Myrtenal	1155	1151	tr	-	MS, RI, AU
30.	Terpene-4-ol	1159	1159	0.93	-	MS, RI
31.	Verbenol	1166	1165	tr	0.87	MS, RI, AU
32.	*α*-Phellandren-8-ol	1167	1167	-	tr	MS, RI
33.	*α*-Terpineol	1169	1169	tr	0.65	MS, RI, AU
34.	Myrtenol	1176	1178	0.59	tr	MS, RI, AU
35.	*n*-Dodecane	1199	1200	tr	0.68	MS, RI
36.	Cuminal	1206	1207	0.34	-	MS, RI
37.	Carveol	1215	1217	tr	-	MS, RI
38.	*trans*-Chrysanthenyl acetate	1243	1243	-	0.42	MS, RI
39.	Isobornyl acetate	1266	1268	0.69	1.23	MS, RI
40.	Carvacrol	1274	1275	tr	-	MS, RI, AU
41.	Thymol	1282	1285	tr	-	MS, RI
42.	(-)-*trans*-Pinocarvyl acetate	1303	1297	0.21	-	MS, RI
43.	*α*-terpinyl acetate	1331	1325	-	0.89	MS, RI
44.	Copaene	1367	1367	tr	5.61	MS, RI
45.	*β*-Elemene	1382	1382	-	1.19	MS, RI
46.	*β* -Patchoulene	1369	1370	0.34	-	MS, RI
47.	*n*-Decanoic acid	1380	1380	tr	-	MS, RI
48.	*n*-Tetradecane	1399	1400	tr	1.27	MS, RI
49.	Caryophyllene	1406	1406	0.32	tr	MS, RI, AU
50.	*β*-Farnesene	1447	1446	-	1.06	MS, RI
51.	α-Curcumene	1467	1464	1.23	10.50	MS, RI
52.	Zingiberene	1486	1486	-	4.33	MS, RI, AU
53.	Varidiflorene	1498	1499	tr	-	MS, RI
54.	*α*-Bisabolene	1500	1500	-	1.37	MS, RI
55.	Guaiene	1506	1500	-	tr	MS, RI
56.	*α*-Muurolene	1511	1508	-	tr	MS, RI
57.	Cadinene	1509	1508	0.3	-	MS, RI
58.	*α*-Calacorene	1523	1523	-	tr	MS, RI
59.	Spathulenol	1535	1537	0.73	-	MS, RI
60.	*trans*-Nerolidol	1548	1549	-	tr	MS, RI
61.	Caryophyllene oxide	1562	1562	5.46	1.27	MS, RI
62.	Isoaromadendrene epoxide	1583	1579	tr	tr	MS, RI
63.	Ent-Spathulenol	1588	1578	1.38	1.27	MS, RI
64.	*n*-Hexadecane	1599	1600	-	tr	MS, RI
65.	Widdrol	1604	1606	-	tr	MS, RI
66.	Cubenol	1608	1609	-	0.56	MS, RI
67.	*α*-Eudesmol	1634	1630	4.4	-	MS, RI
68.	*τ*-Eudesmol	1637	1638	-	8.92	MS, RI
69.	*α*-Cadinol	1651	1651	-	1.05	MS, RI
70.	Cedren-13-ol, 8-	1664	1668	4.94	0.56	MS, RI
71.	Bisabolol	1665	1667	-	2.26	MS, RI
72.	Germacrone	1691	1691	tr	-	MS, RI
73.	*n*-Octadecane	1799	1800	0.54	1.06	MS, RI
74.	Hexahydrofarnesyl acetone	1827	1832	0.30	tr	MS, RI
75.	Nonacosane	1903	1900	2.75	0.75	MS, RI
76.	Palmitic acid, methyl ester	1910	1910	0.24	-	MS, RI
77.	Palmitic acid	1951	1951	0.66	1.54	MS, RI, AU
78.	*n*-Eicosane	1998	2000	0.38	1.32	MS, RI
79.	Methyl linoleate	2072	2078	tr	-	MS, RI
80.	Methyl oleate	2082	2082	tr	-	MS, RI
81.	Heneicosane	2103	2100	tr	4.88	MS, RI
82.	Methyl linolenate	2113	2099	0.26	-	MS, RI
83.	Linoleic acid	2118	2113	tr	-	MS, RI, AU
84.	Tricosane	2304	2300	0.32	4.18	MS, RI
85.	Tetracosane	2397	2400	0.29	1.34	MS, RI
86.	Pentacosane	2503	2500	0.19	8.65	MS, RI
87.	Hexacosane	2605	2600	-	0.29	MS, RI
88.	Heptacosane	2705	2700	-	1.43	MS, RI
89.	Octacosane	2799	2800	-	0.94	MS, RI
Monoterpene hydrocarbons				11.81	2.18	
Oxygenated monoterpens				54.86	27.65	
Sesquiterpene hydrocarbons				2.19	24.06	
Oxygenated sesquiterpens				16.91	15.89	
Others				5.93	29.20	
Total identified components				91.70	98.98	

**Table 2:** Minimum inhibitory concentrations (MIC) in (µg/mL) and mean diameter of inhibition zones (DIZ) in (mm) of *C. indicum* and *C. morifolium* essential oils against different pathogens determined by microdilution and agar diffusion method.

Microorganisms	*C. indicum*		*C. morifolium*		Positive Control *	
	DIZ (mm)	MIC (µg/mL)	DIZ (mm)	MIC (µg/mL)	DIZ (mm)	MIC (µg/mL)
**Gram Positive Bacteria**						
*Bacillus subtilis* ATCC 6051	20.0 ± 0.2	62.5	15.0 ± 0.7	125	31.6 ± 0.3	0.08
*Staphylococcus aureus* ATCC 29213	20.4 ± 0.5	250	19.5 ± 0.3	250	30.1 ± 0.6	0.16
*Staphylococcus capitis* ATCC 35661	19.9 ± 0.4	125	16.1 ± 0.5	250	29.2 ± 0.1	0.08
*Staphylococcus epidermidis* ATCC 14990	19.6 ± 0.7	250	17.4 ± 0.8	250	26.4 ± 0.2	0.16
*Streptococcus agalactiae* ATCC 27956	19.2 ± 0.8	62.5	14.4 ± 0.6	125	30.1 ± 0.1	0.08
*Streptococcus pyogenes* ATCC 12344	21.9 ± 0.9	62.5	17.5 ± 0.3	125	29.7 ± 0.0	0.08
**Gram Negative Bacteria**						
*Escherichia coli* ATCC 25922	13.8 ± 0.2	>500	13.7 ± 0.8	500	32.1 ± 0.0	0.06
*Pseudomonas fluorescens* ATCC 13525	12.3 ± 0.1	>500	11.7 ± 0.1	>500	27.3 ± 0.1	0.24
*Salmonella typhimurium* ATCC 14028	12.1 ± 0.5	>500	11.5 ± 0.1	>500	24.6 ± 0.2	0.96
*Shigella flexneri* ATCC 700930	11.2 ± 0.9	>500	10.9 ± 0.5	>500	21.2 ± 0.0	0.96
**Fungi**						
*Aspergillus fumigatus* ATCC 1022	15.8 ± 0.5	>500	14.5 ± 0.5	500	26.3 ± 0.1	0.12
*Candida albicans* ATCC 90028	13.6 ± 0.7	>500	12.6 ± 0.2	>500	24.8 ± 0.7	0.24
*Geotrichum candidum* ATCC 12784	14.0 ± 0.5	500	13.3 ± 0.4	>500	23.2 ± 0.3	0.48
*Syncephalastrum racemosum* ATCC 14831	11.9 ± 0.6	>500	11.2 ± 0.7	>500	21.4 ± 0.5	0.48

Data are presented as means ± S.D. *n* = 3; * The positive control was taken as ampicillin for Gram-positive bacteria, gentamycin for Gram-negative bacteria and clotrimazole for fungi; all assays consisted of 30 mg essential oil in 1 mL DMSO, and 100 μL were applied. DIZ, diameter of inhibition zone is measured in (mm) by the agar diffusion method.
